# Diabetes-Related Distress, Psychosocial Predictors, and Glycemic Control in Jordanian Adults: A Cross-Sectional Study

**DOI:** 10.7759/cureus.108361

**Published:** 2026-05-06

**Authors:** Enas F Younis, Aws A Ghraiz, Zaid I Qolaghasi, Mohannad Y Ayyad

**Affiliations:** 1 Internal Medicine/Endocrinology, Istishari Hospital, Amman, JOR; 2 Internal Medicine, Hashemite University, Amman, JOR; 3 Internal Medicine, Istishari Hospital, Amman, JOR

**Keywords:** diabetes mellitus, diabetes-related distress, glycated hamoglobin a1c (hba1c), glycemic control, self-management, social support

## Abstract

Background: Diabetes-related distress (DRD) represents a significant emotional burden for individuals living with diabetes mellitus; however, its relationship with glycemic control has received comparatively limited attention in clinical populations across the Middle East. The present study investigated the association between DRD and glycated hemoglobin (HbA1c), alongside the contributory roles of self-management adherence, social support, and self-efficacy.

Methods: We conducted a cross-sectional study of 201 adults with diabetes. DRD was measured using a seven-item Likert scale (1-6), with higher scores indicating greater distress. We also assessed social support (seven items), self-efficacy (five items), and self-management adherence across six behavioral domains. Glycemic control was assessed by HbA1c, with a value below 7% considered adequate. Pearson correlations and multiple linear regression were used to analyze the data.

Results: The mean HbA1c was 7.93 ± 2.01%; only 36.8% of participants reached adequate glycemic control. DRD was positively correlated with HbA1c (r = 0.344, p < 0.001), while self-management adherence (r = −0.313, p < 0.001), social support (r = −0.180, p = 0.011), and self-efficacy (r = −0.143, p = 0.043) were each inversely associated with it.

Participants in the high DRD group had a mean HbA1c of 8.83%, compared with 7.15% in the low DRD group. The regression model explained 24.2% of the variance in HbA1c (R² = 0.242, adjusted R² = 0.219), with DRD emerging as the strongest positive predictor (β = 0.313) and self-management adherence as the strongest negative predictor (β = −0.295); diabetes duration also made a significant independent contribution (β = 0.208, p = 0.002). No significant gender difference in DRD was observed (p = 0.429).

Conclusions: Diabetes-related distress was the strongest independent predictor of poor glycemic control, with self-management adherence and diabetes duration also making significant independent contributions. Social support and self-efficacy showed bivariate associations with HbA1c that were not sustained after multivariate adjustment, suggesting their effects operate indirectly through distress and self-management pathways rather than as direct glycemic predictors. These findings support integrating routine distress screening and multidisciplinary psychosocial care into outpatient diabetes management.

## Introduction

Diabetes mellitus (DM) ranks among the most common chronic metabolic disorders worldwide. More than 537 million adults are currently affected, with projections suggesting this figure will exceed 780 million by 2045 [1]. Although pharmacological treatments have advanced considerably, a large proportion of patients still do not achieve recommended glycemic targets [2], with HbA1c levels remaining at or above 7%. These individuals remain at elevated risk of developing both microvascular and macrovascular complications, including retinopathy, nephropathy, neuropathy, and cardiovascular disease.

Biomedical factors such as medication adherence and coexisting conditions have long been recognized as determinants of glycemic control [2], but the psychosocial dimensions of diabetes care have received increasing attention in recent years. Diabetes-related distress (DRD) describes the negative emotional burden that arises specifically from living with and managing diabetes, including feelings of worry, frustration, anxiety, a sense of losing control, and emotional exhaustion [3,4]. It is not the same as clinical depression; DRD is conceptually distinct and is widely regarded as an inherent emotional response to the unrelenting demands of diabetes self-care [5].

Global prevalence estimates for DRD range from 18% to 45%, depending on the type of diabetes, its duration, and the cultural setting [6]. Elevated DRD has been linked to poorer medication adherence, less frequent self-monitoring of blood glucose, unhealthy eating patterns, and reduced physical activity, all of which contribute to worse glycemic outcomes [7]. DRD, however, does not act in isolation. Grounded in Social Cognitive Theory [8], DRD may act as a proximal barrier that simultaneously erodes self-efficacy and weakens social support, two psychosocial resources widely recognized as buffers against the harmful effects of distress on health behavior [9]. This framework supports the simultaneous examination of DRD, self-efficacy, social support, and self-management adherence as interrelated determinants of glycemic control.

While much of the evidence base comes from Western populations, relatively few studies have examined how DRD, psychosocial mediators, and glycemic outcomes interact in Middle Eastern clinical samples, where cultural, familial, and healthcare system factors may shape these relationships in distinctive ways. Accordingly, this study aimed to: (1) describe the prevalence and severity of DRD in Jordanian adults with diabetes; (2) quantify its association with HbA1c; and (3) assess the independent contributions of self-management adherence, social support, and self-efficacy to glycemic control in this clinical sample.

## Materials and methods

Study design and setting

We conducted a cross-sectional, observational study at a diabetes outpatient clinic at Istishari Hospital, Amman, Jordan. Participants were recruited consecutively between April 2025 and March 2026. Ethical approval was granted by the Institutional Review Board of Istishari Hospital (Approval No. IRBIH/EY/10/04/2025/2), and all participants provided written informed consent prior to enrolment. The study was conducted in accordance with the ethical principles of the Declaration of Helsinki.

The Combined Research Form (CRF) was administered in Arabic, the primary language of the study population, using a validated Arabic adaptation; the English version is provided in Appendices. A trained medical secretary was available to assist participants with clarification of questionnaire items where needed.

Participants

We included adults aged 18 years or older with a confirmed diagnosis of type 1 or type 2 diabetes mellitus. In total, 201 participants were enrolled between April 2025 and March 2026. Participants with cognitive impairment that would prevent questionnaire completion, those actively hospitalized for psychiatric reasons, and pregnant individuals were excluded. Demographic information collected included age, gender, level of education, employment status, and diabetes duration.

Measures

DRD was assessed using seven items adapted from the Diabetes Distress Scale (DDS) [10], covering worry about complications, emotional burden, frustration, perceived inadequate control, lack of familial support, anxiety, and overall management burden. Each item was rated on a six-point Likert scale (1 = not a problem; 6 = a very serious problem), and a composite mean score was calculated, with higher values reflecting greater distress (Cronbach’s α = 0.82).

Social support was measured using seven items adapted from the Multidimensional Scale of Perceived Social Support (MSPSS) [11], assessing perceived emotional support from family and friends. Items were rated on a seven-point Likert scale (1 = strongly disagree; 7 = strongly agree), with higher scores indicating greater perceived support (Cronbach’s α = 0.84).

Self-efficacy was assessed using five items from the General Self-Efficacy Scale (GSES) [12], rated on a four-point scale (1 = not at all true; 4 = exactly true), with higher scores reflecting greater confidence in managing diabetes-related challenges (Cronbach’s α = 0.78). The five-item, four-point format reflects the standard validated structure of the original GSES as developed by Schwarzer and Jerusalem [12] and confirmed across multicultural populations [13].

Self-management adherence was rated across six behavioral domains adapted from the Summary of Diabetes Self-Care Activities (SDSCA) [14], covering the preceding week: healthy diet, physical activity, blood glucose monitoring, medication adherence, stress management, and diabetes education (see the Appendices). Responses were recorded in days per week (0-7), and a mean score was computed across the six domains. Domain-level descriptive statistics are reported separately in the Results to allow examination of variation across individual behavioral areas.

Glycemic control was captured using the most recent HbA1c value (%) drawn either from the medical record or from clinic testing at the time of the visit (source recorded on the CRF for each participant). Adequate glycemic control was defined as HbA1c < 7.0%.

All instruments were administered in Arabic. Items were translated from the original validated source instruments [10-12,14] using standard forward-back translation procedures, with pilot testing conducted prior to main data collection to confirm item clarity and cultural equivalence. The SDSCA was administered using the Arabic adaptation previously applied in a Jordanian clinical sample [15].

Statistical analysis

All statistical analyses were performed using Python (v3.12) with the pandas [16], NumPy [17], and SciPy [18] libraries. Descriptive statistics, including means, standard deviations, and frequencies, were computed for all study variables (N = 201).

Prior to regression modeling, interscale Pearson correlations among all four psychosocial measures (DRD, social support, self-efficacy, and self-management adherence) were computed to evaluate discriminant validity and to confirm the absence of multicollinearity among predictors (Table 1). Variance inflation factors (VIF) were calculated for all regression predictors as an additional multicollinearity check. Pearson correlation coefficients were then used to examine bivariate associations between each psychosocial scale and HbA1c. Spearman rank correlations were additionally computed to assess ordinal trend consistency.

**Table 1:** Interscale Pearson Correlations Among Psychosocial Measures (N = 201) Discriminant validity threshold: |r| < 0.50 = acceptable; |r| ≥ 0.70 = poor. Variance inflation factors ranged from 1.084 to 1.381, confirming no multicollinearity among predictors. r: Pearson correlation coefficient; p: two-tailed significance; DRD: diabetes-related distress; SS: social support; SE: self-efficacy; SM: self-management adherence.

Scale Pair	r	p	Discriminant Validity
DRD × SS	−0.280	<0.001	Acceptable
DRD × SE	−0.215	0.002	Acceptable
DRD × SM	−0.088	0.215	Acceptable
SS × SE	+0.454	<0.001	Acceptable
SS × SM	+0.272	<0.001	Acceptable
SE × SM	+0.172	0.015	Acceptable

For group comparisons, an independent-samples t-test was used to compare mean DRD scores between participants with adequate (HbA1c < 7%) and inadequate (HbA1c ≥ 7%) glycemic control. DRD severity was further classified into three mutually exclusive categories: low (< 2.0), moderate (2.0-<4.0), and high (≥ 4.0) based on published cut-points [19]. Differences in mean HbA1c across the three DRD severity groups were examined using a one-way analysis of variance (ANOVA), with Tukey’s Honest Significant Difference (HSD) post hoc test applied to identify statistically significant pairwise differences. Effect size was quantified using eta squared (η²), with η² ≥ 0.06 indicating a medium effect and η² ≥ 0.14 indicating a large effect [20]. The distribution of adequate glycemic control (HbA1c < 7%) across DRD severity groups was examined using a chi-square test of independence.

Normality of the outcome variable (HbA1c) and regression residuals was assessed using the Shapiro-Wilk test prior to modeling. A multiple linear regression model was then constructed with HbA1c as the dependent variable and DRD, social support (SS), self-efficacy (SE), self-management adherence (SM), age, and diabetes duration as independent predictors. Both the unadjusted coefficient of determination (R²) and adjusted R² are reported to account for the number of predictors in the model. Unstandardized (B) and standardized (β) regression coefficients are presented alongside their standard errors, t-statistics, and 95% confidence intervals. Complete-case analysis was applied; the overall missing data rate was below 1% across all variables, and no imputation was performed. Statistical significance was defined as a two-tailed p-value < 0.05.

A pre-specified sensitivity analysis was conducted by adding diabetes type (type 1 vs. type 2) as a covariate to the regression model to assess whether the primary findings were consistent across diabetes types.

Pre-specified secondary analysis: Prior to data collection, comparison of DRD scores between male and female participants was designated as a secondary objective to examine potential gender differences in diabetes-related distress.

## Results

Sample characteristics

In total, 201 participants were included in the analysis (Table 2). The sample was predominantly type 2 diabetes (n = 173, 86.1%), with 28 participants (13.9%) having type 1 diabetes. The mean age was 56.0 ± 15.4 years, with a roughly equal gender split comprising 101 males (50.2%) and 100 females (49.8%), and a mean diabetes duration of 10.1 ± 8.3 years. Just over half of participants (n = 113, 56.2%) had reached tertiary education. The overall mean HbA1c was 7.93 ± 2.01%, and 127 (63.2%) participants fell into the poor glycemic control category (HbA1c ≥ 7%). 

**Table 2:** Sociodemographic and Clinical Characteristics of Study Participants (N = 201) SD: standard deviation.

Characteristics	n (%) or Mean ± SD	
Total	201 (100%)	
Age (years)	56.0 ± 15.4	
Gender		
Male	101 (50.2%)	
Female	100 (49.8%)	
Type of diabetes		
Type 1	28 (13.9%)	
Type 2	173 (86.1%)	
Diabetes duration (years)	10.1 ± 8.3	
Education level		
Tertiary education	113 (56.2%)	
Secondary education	57 (28.4%)	
Primary/no formal education	31 (15.4%)	
Employment status		
Employed	87 (43.3%)	
Unemployed	63 (31.3%)	
Retired	46 (22.9%)	
Student	5 (2.5%)	
HbA1c (%)	7.93 ± 2.01	
Glycemic control		
Adequate control (HbA1c < 7%)	74 (36.8%)	
Inadequate control (HbA1c ≥ 7%)	127 (63.2%)	

Psychosocial scale scores

Mean scores across the psychosocial instruments are summarized in Table 3. The mean DRD score was 3.20 ± 1.31 on a 1-6 scale, indicating a moderate overall level of distress in the sample. When grouped by DRD category, 40 participants (19.9%) fell into the low distress range, 102 (50.7%) into moderate distress, and 59 (29.4%) into high distress. Social support was at a moderate-to-high level (mean 4.36 ± 1.45), and self-management adherence was also moderate (mean 4.14 ± 1.40 days/week). Self-efficacy, by contrast, was relatively low (mean 2.80 ± 0.63 on a 1-4 scale), consistent with evidence that sustained distress erodes confidence in self-management capacity. Domain-level inspection of the SDSCA revealed substantial variation in adherence across behaviors: medication adherence was the highest-rated domain (M = 5.91, SD = 1.83 days/week), while diabetes education and healthcare contact were the lowest (M = 0.85, SD = 1.52 days/week). This suggests that while participants were generally adherent to medication regimens, engagement with education and structured diabetes care activities was markedly limited. 

**Table 3:** Descriptive Statistics for Psychosocial Scales SD: standard deviation; α: internal consistency estimate. Cronbach’s α is not reported for the Summary of Diabetes Self-Care Activities (SDSCA) composite because its six domains represent distinct behavioral indicators rather than a unitary latent construct.

Scale	Mean	SD	Range	Cronbach's α
Diabetes-related distress (DRD)	3.20	1.31	1–6	0.82
Social support	4.36	1.45	1–7	0.84
Self-efficacy	2.80	0.63	1–4	0.78
Self-management adherence	4.14	1.40	Days/week (0–7)	N/A

Bivariate associations with HbA1c

Pearson correlation analysis showed that DRD had a statistically significant, moderate positive association with HbA1c (r = 0.344, p < 0.001), indicating that higher diabetes-related distress was associated with poorer glycemic control. Conversely, self-management adherence (r = −0.313, p < 0.001), social support (r = −0.180, p = 0.011), and self-efficacy (r = −0.143, p = 0.043) were each inversely and significantly associated with HbA1c (Table 4). 

**Table 4:** Pearson Correlations Between Psychosocial Variables and HbA1c * p < 0.05; *** p < 0.001 DRD: diabetes-related distress.

Variable	r	p-value	Direction	Significance
DRD score → HbA1c	0.344	< 0.001	Positive	***
Self-management → HbA1c	−0.313	< 0.001	Negative	***
Social support → HbA1c	−0.180	0.011	Negative	*
Self-efficacy → HbA1c	−0.143	0.043	Negative	*

HbA1c by DRD category

Mean HbA1c increased monotonically across DRD severity categories (Table 5). Participants in the low-, moderate-, and high-DRD groups had mean HbA1c values of 7.15% (SD = 1.55), 7.72% (SD = 1.85), and 8.83% (SD = 2.23), respectively, representing a clinically meaningful difference of 1.68 percentage points between the extreme groups.

**Table 5:** Diabetes-Related Distress (DRD) Levels and Glycemic Control (N = 201) F-statistic, η², and p-value are reported on the Low DRD row; dashes indicate these statistics apply to the overall ANOVA and not to individual group rows. One-way ANOVA compared mean HbA1c across the three DRD severity groups (Low, Moderate, High). η² = 0.095 indicates a medium-to-large effect size [20]. Controlled glycemia defined as HbA1c < 7.0%. DRD scored on a 1–6 scale (mean of 7 items). *** p < 0.001.

DRD Group	n (%)	HbA1c Mean (%)	HbA1c SD	Controlled <7% n (%)	F-Statistic	η²	p-value
Low DRD (< 2)	40 (19.9%)	7.15	1.55	20 (50.0%)	F = 10.451	0.095	< 0.001***
Moderate DRD (2–<4)	102 (50.7%)	7.72	1.85	41 (40.2%)	—	—	—
High DRD (4–6)	59 (29.4%)	8.83	2.23	13 (22.0%)	—	—	—
Total	201 (100%)	7.93	2.01	74 (36.8%)	—	—	—

A one-way ANOVA confirmed a statistically significant difference in mean HbA1c across the three DRD groups (F(2, 198) = 10.451, p < 0.001, η² = 0.095), representing a medium-to-large effect size [20]. Tukey’s HSD post hoc comparisons revealed that the high-DRD group had significantly higher HbA1c than both the low-DRD group (mean difference = 1.684%, q = 6.066, p < 0.001) and the moderate-DRD group (mean difference = 1.108%, q = 4.999, p = 0.002). The difference between the low- and moderate-DRD groups did not reach statistical significance (mean difference = 0.576%, q = 2.277, p = 0.243). Linear trend analysis confirmed a significant monotonic association between DRD severity and HbA1c using both Pearson correlation (r = 0.302, p < 0.001) and Spearman rank correlation (ρ = 0.322, p < 0.001) (Table 6).

**Table 6:** Post-Hoc Comparisons, Linear Trend and Proportion Tests B1 Tukey HSD: q-statistic (studentized range, k = 3 groups, df = 198); p-values adjusted for multiple comparisons. Negative mean differences indicate lower HbA1c in the first-named group. B2 Trend: low = 1, moderate = 2, high = 3 (ordinal coding). B3 Chi-square: 2 df; compares proportions achieving HbA1c < 7% across the three DRD groups. ** p < 0.01; *** p < 0.001. All p-values are two-tailed.

Comparison / Test	Statistic	Value	p-value
B1 Tukey HSD post-hoc pairwise comparisons (mean HbA1c difference between groups)			
Low vs. moderate	Mean diff.	−0.576% (q = 2.277)	0.243 (ns)
Low vs. high	Mean diff.	−1.684% (q = 6.066)	<0.001***
Moderate vs. high	Mean diff.	−1.108% (q = 4.999)	0.002**
B2 linear trend (ordinal DRD category × continuous HbA1c)			
Linear trend	Pearson r	r = 0.302	<0.001***
Linear trend	Spearman ρ	ρ = 0.322	<0.001***
B3 controlled glycemia proportions across DRD groups			
Low 50.0% / Moderate 40.2% / High 22.0%	χ²(2)	9.032	0.011*

The proportion of participants achieving adequate glycemic control (HbA1c < 7%) decreased significantly across DRD severity groups: 50.0% in the low-DRD group (20/40), 40.2% in the moderate-DRD group (41/102), and 22.0% in the high-DRD group (13/59), as confirmed by a chi-square test of independence (χ²(2) = 9.032, p = 0.011). 

Regression diagnostics

Residual normality was assessed using the Shapiro-Wilk test (W = 0.912, p < 0.001). Although statistically significant, visual inspection of the Q-Q plot confirmed approximate normality with no severe departures, supporting the robustness of OLS regression at this sample size (N = 201). Homoscedasticity was confirmed using the Breusch-Pagan test (LM = 6.01, p = 0.42), indicating that residual variance was constant across predicted values. VIF scores ranged from 1.084 (self-management adherence) to 1.381 (social support), all well below the threshold of 5.0, confirming the absence of multicollinearity among predictors.

Multiple regression analysis

The multiple linear regression model (Table 7) demonstrated significant overall fit (F(6, 194) = 10.323, p < 0.001), accounting for 24.2% of the variance in HbA1c (R² = 0.242, adjusted R² = 0.219). This indicates that approximately 75.8% of HbA1c variance remains unexplained by the psychosocial predictors in this model, reflecting the substantial contribution of biological and pharmacological factors not captured here. DRD was the strongest independent predictor (β = 0.313, the largest standardized coefficient in the model), with higher distress significantly associated with poorer glycemic control (B = 0.478, SE = 0.100, t = 4.779, p < 0.001, 95% CI (0.281, 0.676)).

Lower self-management adherence was also a significant independent predictor of higher HbA1c (B = −0.475, β = −0.295, SE = 0.105, t = −4.520, p < 0.001, 95% CI (−0.682, −0.268)). Longer diabetes duration made a statistically significant positive contribution (B = 0.050, β = 0.208, SE = 0.016, t = 3.125, p = 0.002, 95% CI (0.018, 0.082)).

Social support (B = −0.023, β = −0.017, p = 0.823), self-efficacy (B = −0.093, β = −0.029, p = 0.682), and age (B < 0.001, β = 0.003, p = 0.966) did not independently predict HbA1c after adjustment for all model variables. Ninety-five percent confidence intervals for all unstandardized coefficients are reported in Table 7; all confidence intervals for significant predictors exclude zero, consistent with their p-values.

**Table 7:** Multiple Linear Regression Analysis: Predictors of HbA1c Among Adults With Diabetes (N = 201) B = unstandardized regression coefficient: change in HbA1c (%) per one-unit increase in the predictor, holding all other variables constant. β = standardized regression coefficient: enables direct comparison of relative predictor strength across variables measured on different scales. SE = standard error of B. t = ratio of B to its standard error; tests H₀: B = 0. p = two-tailed significance. 95% CI = confidence interval for the unstandardized coefficient B; CIs not crossing zero are consistent with statistical significance at α = 0.05. Data handling: self-management adherence: three participants (1.5%) recorded responses as ranges (e.g., “4-5 days”); the lower bound was used, which may marginally underestimate adherence for these participants. Diabetes duration: three participants reported duration in months; values were converted using 1 month = 0.083 year (1/12), yielding 3 months = 0.25 yr and 6 months = 0.50 yr. No data were excluded or imputed. Model fit: R² = 0.242, adjusted R² = 0.219, F(6, 194) = 10.323, p < 0.001, N = 201. Significance thresholds: ** p < 0.01; *** p < 0.001.

Variable	B	β	SE	t	p-value	95% CI for B
Intercept	7.948	—	0.984	8.077	< 0.001***	(6.008, 9.889)
Diabetes-related distress mean score	0.478	0.313	0.100	4.779	< 0.001***	(0.281, 0.676)
Self-management adherence	−0.475	−0.295	0.105	−4.520	< 0.001***	(−0.682, −0.268)
Social support	−0.023	−0.017	0.103	−0.224	0.823	(−0.225, 0.179)
Self-efficacy	−0.093	−0.029	0.227	−0.410	0.682	(−0.541, 0.355)
Age (years)	0.0004	0.003	0.009	0.043	0.966	(−0.017, 0.018)
Diabetes duration (years)	0.050	0.208	0.016	3.125	0.002**	(0.018, 0.082)

Sensitivity analysis

When diabetes type was added as a covariate, DRD (B = 0.452, p < 0.001) and self-management adherence (B = −0.488, p < 0.001) remained the strongest independent predictors. Diabetes type was not a significant predictor (B = −0.308, p = 0.586, with type 1 as the reference category), confirming that findings were consistent across type 1 and type 2 diabetes.

Gender differences in DRD

As a pre-specified secondary analysis, DRD scores were compared between male and female participants. There was no statistically significant difference in DRD scores between males (mean = 3.12, SD = 1.34, n = 101) and females (mean = 3.27, SD = 1.29, n = 100; t = −0.793, p = 0.429), indicating that diabetes-related distress was distributed similarly across genders in this sample. DRD scores also showed no meaningful correlation with age (r = −0.001, p = 0.986) or diabetes duration (r = −0.041, p = 0.564), indicating that DRD severity was not influenced by how long participants had lived with diabetes. Importantly, this finding is distinct from the relationship between diabetes duration and HbA1c: although longer disease duration was not associated with greater DRD, it was a significant independent predictor of poorer glycemic control in the regression model (β = 0.208, p = 0.002).

## Discussion

This cross-sectional study demonstrates that diabetes-related distress is a clinically meaningful and statistically significant predictor of glycemic control in a Jordanian clinical population. The central finding is a moderate positive correlation between DRD and HbA1c (r = 0.344, p < 0.001), which aligns with existing literature and strengthens the case for integrating psychological assessment into routine diabetes care [21].

The overall mean HbA1c of 7.93% and the fact that 63.2% of participants had inadequate glycemic control indicate a substantial unmet clinical need. Participants in the high-DRD category had a mean HbA1c of 8.83%, almost 1.68 percentage points higher than those in the low-DRD group (7.15%). This difference is clinically significant, as each 1% reduction in HbA1c has been linked to a 21% decrease in diabetes-related deaths and substantial reductions in microvascular complications [22]. The ANOVA effect size (η² = 0.095) confirms a medium-to-large magnitude of association between DRD severity and glycemic control.

The inverse association between self-management adherence and HbA1c (r = −0.313) aligns with the well-established finding that steady engagement in health behaviors, including diet, physical activity, medication use, and glucose monitoring, is fundamental to achieving glycemic control [23]. This pattern is consistent with prior longitudinal evidence suggesting that DRD may erode these self-care behaviors through emotional exhaustion, reduced motivation, and cognitive preoccupation with the burden of disease [23]. The standardized regression coefficients provide effect size context: β = 0.313 for DRD and β = −0.295 for self-management adherence represent moderate standardized effects of comparable clinical importance.

The DRD-HbA1c association is further supported by a plausible neuroendocrine mechanism. Chronic psychological stress activates the hypothalamic-pituitary-adrenal (HPA) axis, triggering sustained elevations in cortisol and counterregulatory hormones, including glucagon and catecholamines. These hormones directly impair insulin sensitivity and stimulate hepatic glucose output, contributing to hyperglycemia through a pathway that operates independently of behavioral self-care [24]. This biological mechanism is consistent with evidence that progressive beta-cell dysfunction in long-standing diabetes further impairs the metabolic response to psychological stress [25]. Taken together, the behavioral and neuroendocrine pathways offer a convergent explanation for the DRD-HbA1c association: distress simultaneously undermines self-care behavior and directly perturbs the physiological mechanisms of glucose homeostasis.

Social support emerged as a significant negative predictor of HbA1c in bivariate analysis (r = −0.180, p = 0.011). The role of family and wider social networks in sustaining diabetes self-management is well documented: family-integrated education programs have been shown to improve medication adherence, glycemic control, and psychological well-being in patients with uncontrolled type 2 diabetes [26]. However, social support did not retain independent significance in the multivariate regression model (β = −0.017, p = 0.823). This attenuation is consistent with path-analytic evidence showing that the association between social support and HbA1c is mediated through psychological and motivational intermediaries rather than operating as a direct glycemic effect [27]. Similarly, self-efficacy was inversely associated with HbA1c in bivariate analysis (r = −0.143, p = 0.043) but did not independently predict HbA1c after adjustment (β = −0.029, p = 0.682). These findings are theoretically coherent: within the Social Cognitive Theory framework adopted in this study, social support and self-efficacy may exert their effects on glycemic outcomes indirectly, primarily through their buffering influence on distress levels and their facilitation of self-management adherence, rather than constituting independent glycemic predictors in their own right. Future mediation analyses are warranted to test these pathways explicitly.

Importantly, diabetes duration emerged as the third statistically significant independent predictor of HbA1c (B = 0.050, β = 0.208, p = 0.002). This is consistent with the well-established trajectory of progressive beta-cell deterioration and cumulative treatment fatigue in long-standing diabetes [25]. Notably, diabetes duration was not correlated with DRD in this sample (r = −0.041, p = 0.564), consistent with the hypothesis that its contribution to glycemic control may operate through pathways independent of psychological distress, most likely through declining pancreatic reserve and pharmacological factors. Clinically, this finding reinforces the need for intensified glycemic monitoring and proactive care as disease duration increases, over and above the management of psychological distress.

The full regression model (R² = 0.242) accounts for 24.2% of HbA1c variance. The remaining 75.8% is unexplained by the psychosocial predictors in this model, a primary finding that underscores the multidetermined nature of glycemic control. Biological factors not assessed here, including insulin regimen type, beta-cell function, BMI, comorbidity burden, and access to structured diabetes education, are likely to account for a substantial portion of this unexplained variance. Future studies should incorporate these clinical covariates alongside psychosocial measures to achieve a more complete explanatory model of glycemic outcomes.

The low mean self-efficacy score (2.80 ± 0.63) observed in this sample is clinically noteworthy. Within the Social Cognitive Theory framework informing this study, sustained distress erodes confidence in self-management capacity. Interventions targeting DRD may therefore secondarily restore self-efficacy rather than requiring separate efficacy-building programs.

The absence of a significant gender difference in DRD (p = 0.429) and the lack of association with age (r = −0.001, p = 0.986) imply that psychological distress is widespread across demographic subgroups in this sample, supporting the case for universal rather than risk-stratified screening.

This study has several methodological considerations. The cross-sectional design precludes causal inference. The convenience sample from a single outpatient clinic limits generalizability. Self-report methodology is subject to recall and social desirability bias. The small type 1 diabetes subsample (n = 28, 13.9%) was insufficient for meaningful subgroup comparison; future studies should recruit balanced samples. The mixed HbA1c data source may introduce minor inter-assay variability, although a sensitivity analysis confirmed no meaningful difference between source groups. The absence of prospective study registration is also acknowledged.

## Conclusions

Diabetes-related distress emerged as the strongest independent predictor of poor glycemic control in this Jordanian outpatient sample, alongside significant independent contributions from self-management adherence and diabetes duration. The attenuation of social support and self-efficacy in the multivariate model suggests that these psychosocial resources may operate indirectly, most likely through their relationship with distress levels and self-care behaviors, rather than exerting direct effects on glycemic outcomes. Future mediation analyses are warranted to test this hypothesis explicitly.

These findings carry clear clinical implications. Routine validated screening for diabetes-related distress should be embedded into outpatient diabetes care, using instruments such as the Diabetes Distress Scale or the Problem Areas in Diabetes (PAID) scale. Multidisciplinary care models that address emotional burden alongside pharmacological management are warranted, and interventions targeting self-management adherence remain a central priority given the independent contribution of self-management adherence to glycemic control. The low self-efficacy observed in this sample further suggests that distress-reduction programs may secondarily restore patients’ confidence in self-care, without requiring separate efficacy-building components.

Longitudinal and interventional research is needed to determine whether targeted reduction of diabetes-related distress translates into sustained improvements in glycemic control, and to clarify the mediating pathways through which social support and self-efficacy influence glycemic outcomes in Middle Eastern clinical populations.

## Appendices

**Table 8:** Combined Research Form (CRF): Diabetes-Related Distress, Psychosocial Predictors, and Glycemic Control Study All scales used validated Arabic adaptations. HbA1c values retrieved from medical records or same-day clinic testing (source documented per participant).

S. No.	Item	Response
Section 1: General Information (7 items: demographic & clinical background)		
1	Age (years)	Open numeric entry ____ years
2	Gender	Male Female
3	Type of diabetes	Type 1 Type 2 Other
4	Duration of diabetes diagnosis (years)	Open numeric entry ____ years
5	HbA1c (%)	Extracted from medical record/clinic test ____%
6	Education level	No formal Primary Secondary Tertiary
7	Employment status	Employed Unemployed Retired Student
Section 2: Diabetes Distress Scale (DDS) (7 items): Scale: 1 = Not a problem → 6 = Very serious problem		
8	I worry about the long-term complications of diabetes.	1 2 3 4 5 6
9	I feel overwhelmed by the demands of living with diabetes.	1 2 3 4 5 6
10	I feel frustrated with my diabetes management.	1 2 3 4 5 6
11	I feel unsupported by my family and friends.	1 2 3 4 5 6
12	I feel anxious about my diabetes.	1 2 3 4 5 6
13	I feel that diabetes takes up too much of my mental and physical energy.	1 2 3 4 5 6
14	I feel that I am not in control of my diabetes.	1 2 3 4 5 6
Section 3: Multidimensional Scale of Perceived Social Support (MSPSS) (7 items): Scale: 1 = Strongly disagree → 7 = Strongly agree		
15	I get the emotional help and support I need from my family.	1 2 3 4 5 6 7
16	I have friends who really try to help me.	1 2 3 4 5 6 7
17	There is a special person in my life who cares about my feelings.	1 2 3 4 5 6 7
18	I can count on my friends when things go wrong.	1 2 3 4 5 6 7
19	My family understands my diabetes-related problems.	1 2 3 4 5 6 7
20	I can talk about my problems with my family.	1 2 3 4 5 6 7
21	My friends are supportive when I face challenges with my diabetes.	1 2 3 4 5 6 7
Section 4: Generalized Self-Efficacy Scale (GSES) (5 items): Scale: 1 = Not at all true → 4 = Exactly true		
22	I can always manage to solve difficult problems if I try hard enough.	1 2 3 4
23	If someone opposes me, I can find the means and ways to get what I want.	1 2 3 4
24	I am confident that I could deal efficiently with unexpected events.	1 2 3 4
25	I can always manage to cope with the challenges I face.	1 2 3 4
26	I believe I have the ability to tackle problems that come my way.	1 2 3 4
Section 5: Summary of Diabetes Self-Care Activities (SDSCA) (6 items): Scale: 0–7 days in the past week		
27	In the past week, how many days did you follow a healthy diet?	0 1 2 3 4 5 6 7
28	In the past week, how many days did you engage in physical activity?	0 1 2 3 4 5 6 7
29	In the past week, how many days did you check your blood glucose levels?	0 1 2 3 4 5 6 7
30	In the past week, how many days did you take your diabetes medication as prescribed?	0 1 2 3 4 5 6 7
31	In the past week, how many days did you manage your stress effectively?	0 1 2 3 4 5 6 7
32	In the past week, how many days did you participate in diabetes education or speak with a healthcare provider?	0 1 2 3 4 5 6 7
